# Supplementary material for: Smooth muscle-specific MMP17 (MT4-MMP) regulates the intestinal stem cell niche and regeneration after damage
Source: Nat Commun. 2021 Nov 18;12:6741. doi: 10.1038/s41467-021-26904-6 (PMC8602650; doi:10.1038/s41467-021-26904-6)
Supplement: Supplementary file 1 — Supplementary Information [file 41467_2021_26904_MOESM1_ESM.pdf]

Smooth muscle-specific MMP17 (MT4-MMP) regulates the intestinal stem cell niche and regeneration after damage

Mara Martín-Alonso[1], Sharif Iqbal[2,3], Pia M. Vornewald[1], Håvard T. Lindholm[1], Mirjam J. Damen[4], Fernando Martínez[5,6], Sigrid Hoel[1], Alberto Díez-Sánchez[1], Maarten Altelaar[4], Pekka Katajisto[2,3,7], Alicia G. Arroyo[8,9], Menno J. Oudhoff[1]

[1]Centre of Molecular Inflammation Research, and Department of Clinical and Molecular Medicine, Norwegian University of Science and Technology, Trondheim, Norway

[2]Institute of Biotechnology, HiLIFE, University of Helsinki, Finland

[3]Molecular and Integrative Bioscience Research Programme, Faculty of Biological and Environmental Sciences, University of Helsinki, Helsinki, Finland

[4] Biomolecular Mass Spectrometry and Proteomics, Bijvoet Center for Biomolecular Research and Utrecht Institute for Pharmaceutical Sciences, Utrecht University, Utrecht, Netherlands.

[5] Bioinformatics Unit. Centro Nacional de Investigaciones Cardiovasculares (CNIC), Madrid, Spain.CNIC, Madrid, Spain

[6]Centro de Investigación Biomédica en Red de Enfermedades Cardiovasculares (CIBERCV), Madrid, Spain

[7]Department of Biosciences and Nutrition, Karolinska Institutet, Stockholm, Sweden

[8]Department of Molecular Biomedicine, Centro de Investigaciones Biológicas Margarita Salas (CIB-CSIC), Madrid, Spain

[9]Vascular Pathophysiology Area, Centro Nacional de Investigaciones Cardiovasculares (CNIC), Madrid, Spain.

Correspondence should be addressed to MMA (e-mail: mara.m.alonso@ntnu.no) and MJO (email: menno.oudhoff@ntnu.no).

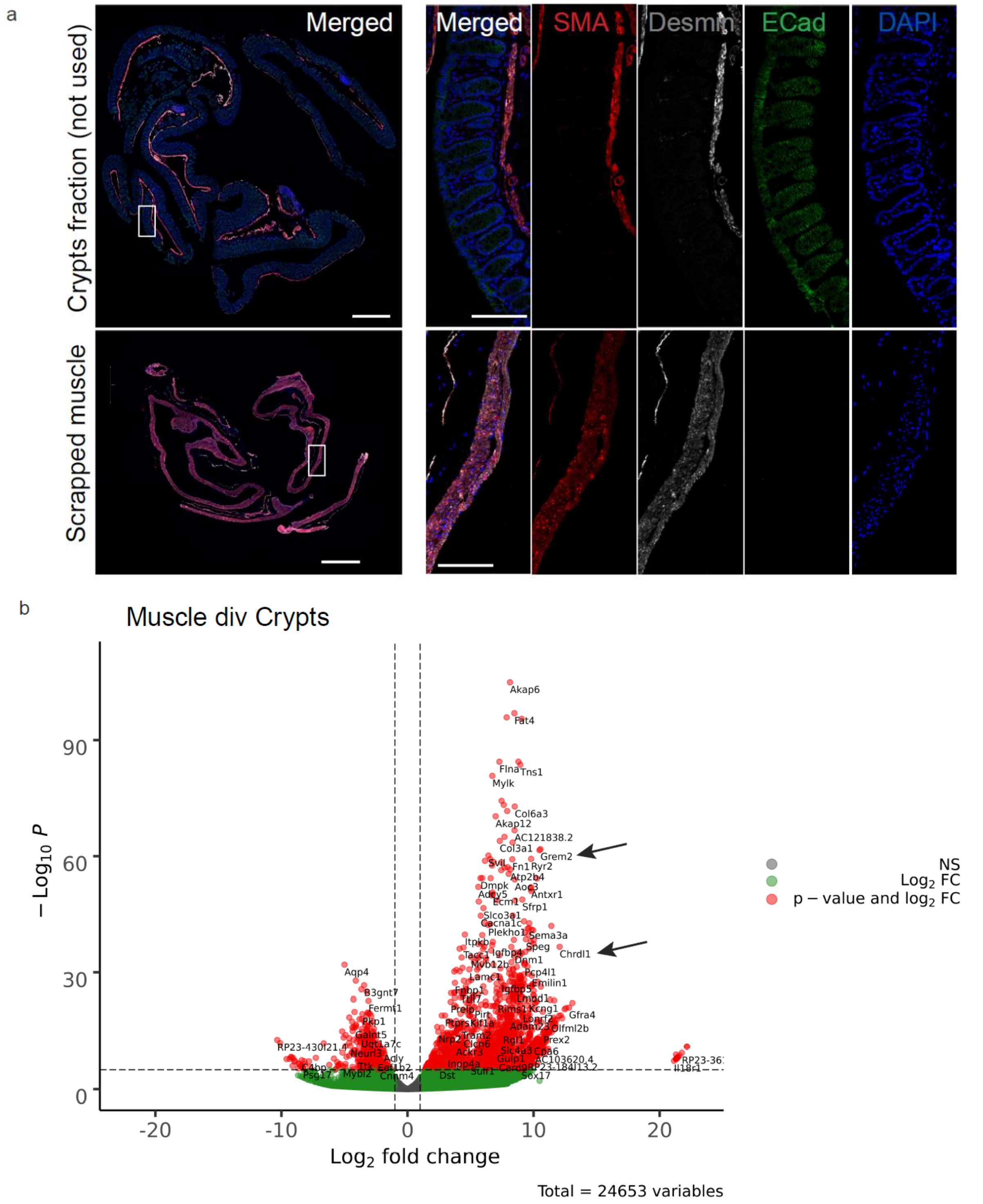

Figure S1. Purity of smooth muscle explants and RNAseq of intestinal crypts. a, Representative confocal image of mucosa fraction (top) and muscle fraction (bottom), showing only Desmin/SMA double positive cells in the muscle fraction (smooth muscle cells) and absence of myofibroblasts (SMA+Desmin- cells). Tissue was stained for SMC markers SMA (red), Desmin (grey), and epithelial marker E-Cadherin (green). Nuclei were stained with DAPI (blue). Only scrapped clean muscle was used for RNAseq or to obtain muscle-SN. Crypts fraction obtained by this method was discarded. Scale in tile scan is 500 µm and 100 µm in insets. n=3 biological replicates **b,** Volcano plot showing differential

expression of genes between muscle and crypts as Gremlins and Chordin like1 (arrows), BMP signaling antagonists. n= 3 biological replicates.

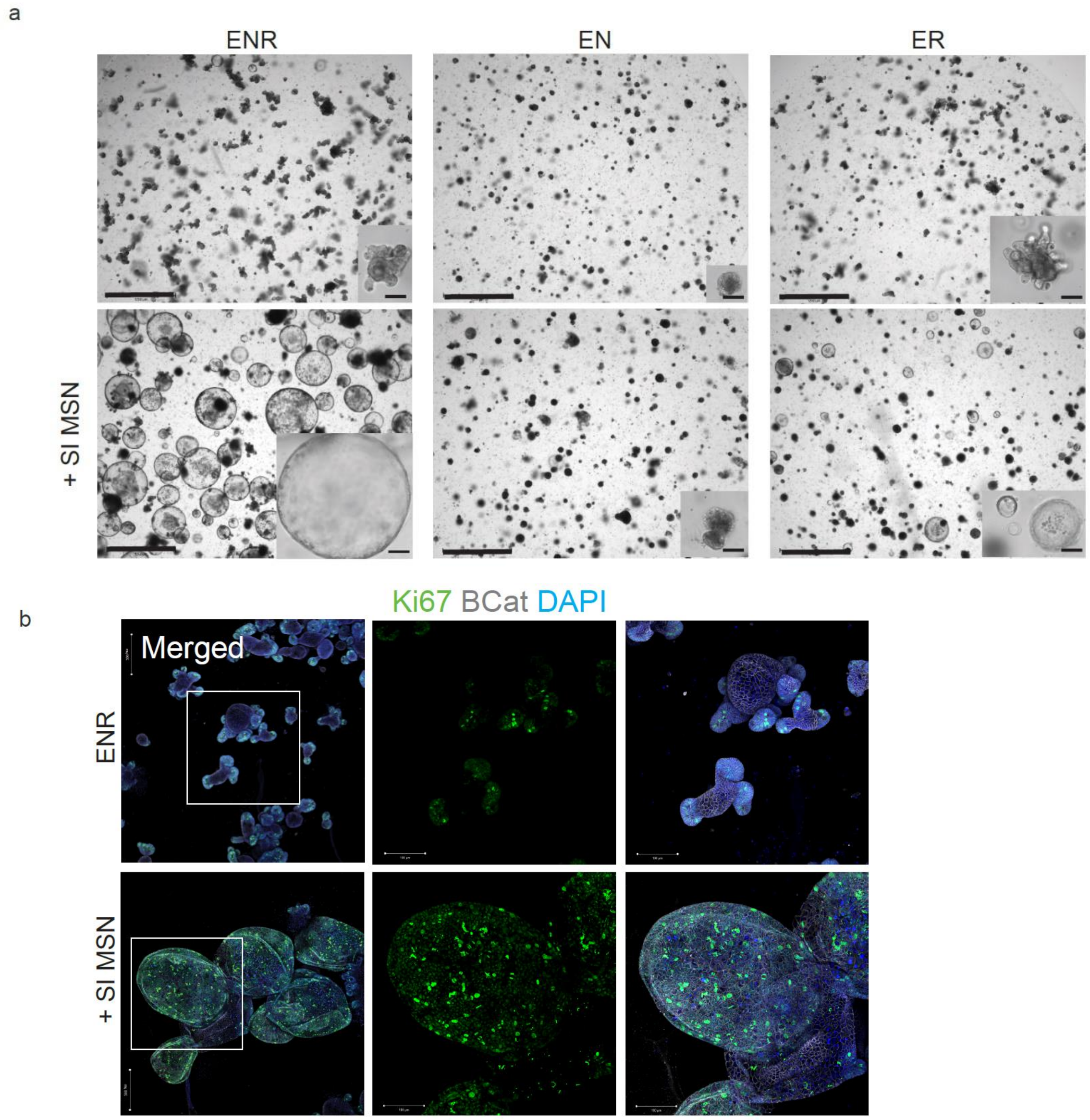

Figure S2. Small intestinal derived muscle also induces large spheroids. a. Small intestinal muscle supernatant (SI MSN) induces large spheroid organoids, similar to colonic muscle supernatant that was used throughout the main manuscript. SI MSN cannot replace RSPO1, but can replace NOGGIN in the culture medium. Scale is 1250 µm and 100 µm on inset. n= 3 wells per condition. 2 independent experiments performed. **b,** Ki67 (green) has crypt-specific staining in ENR organoids, but in SI MSN-mediated large spheroids Ki67 is positive throughout organoid. Scale 200 µm and 100 µm on inset. n= 3 wells per condition. 2 independent experiments performed.

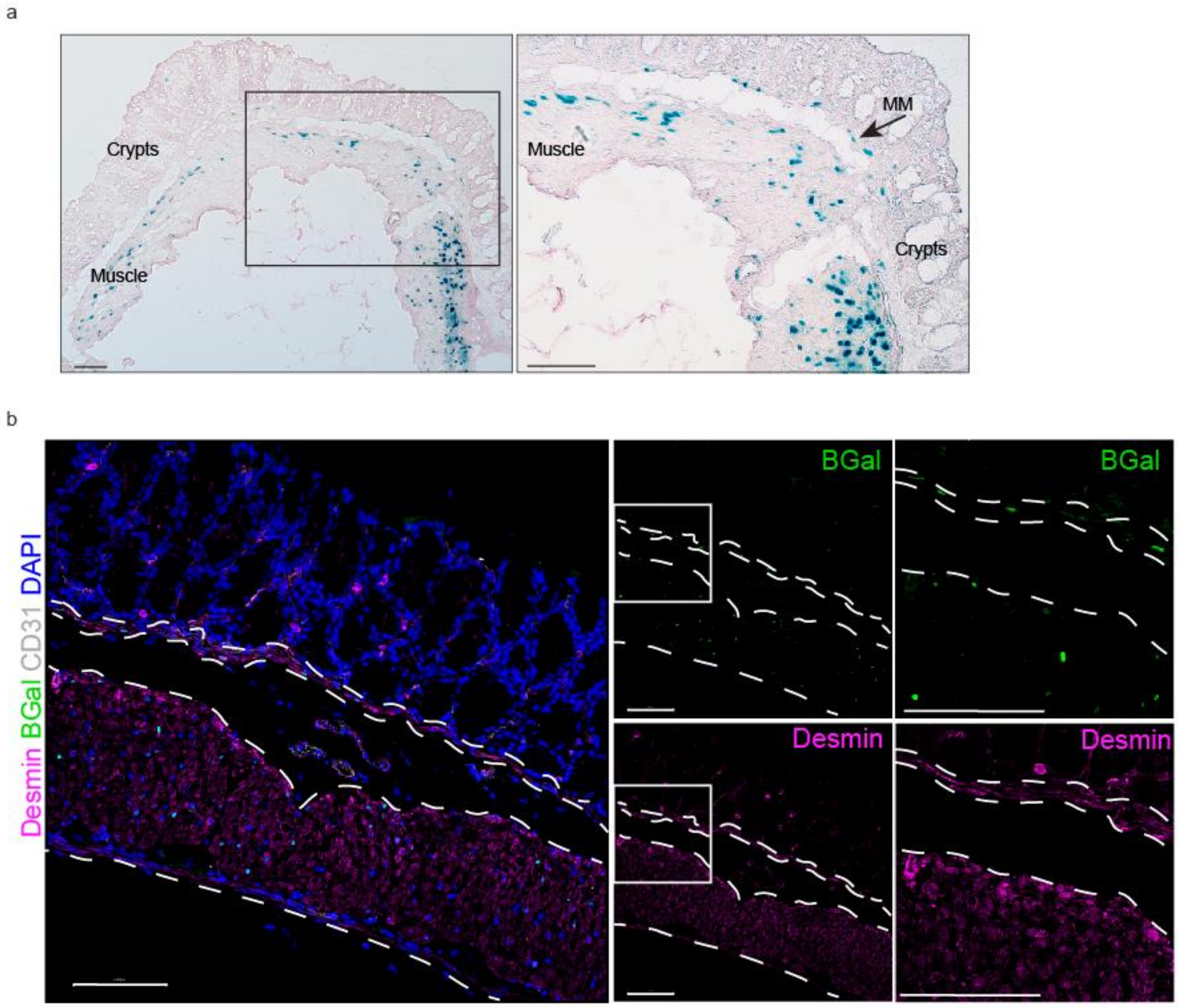

Figure S3. Mmp17 promoter is active in muscle cells from muscularis mucosa and circular and longitudinal muscle. a. Representative image of a transverse colon cut stained for β-Galactosidase activity (blue). Scale bar 100 µm. n= 3 Mmp17+/- mice. **b.** Reprentative images of Desmin (smooth muscle), anti-BGal, CD31 (endothelium) and DAPI stained sections showing that MMP17-BGal positive cells (nuclear staining) are Desmin positive (cytoplasmic) in the smooth muscle. Muscle is highlighted by a dashed white line. Scale bar 100 um. n= 3 Mmp17+/- mice.

a

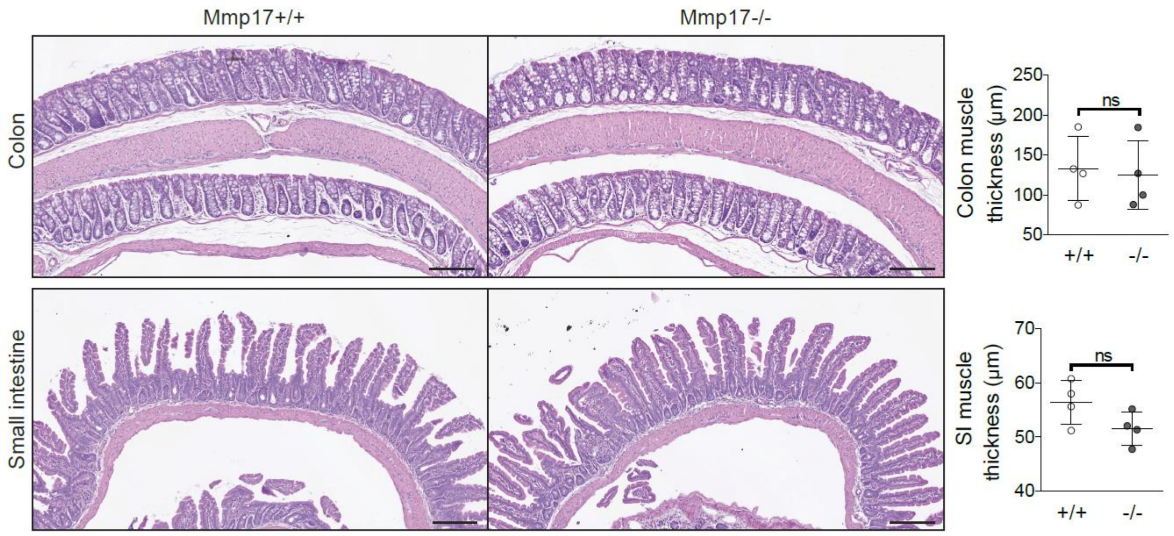

b

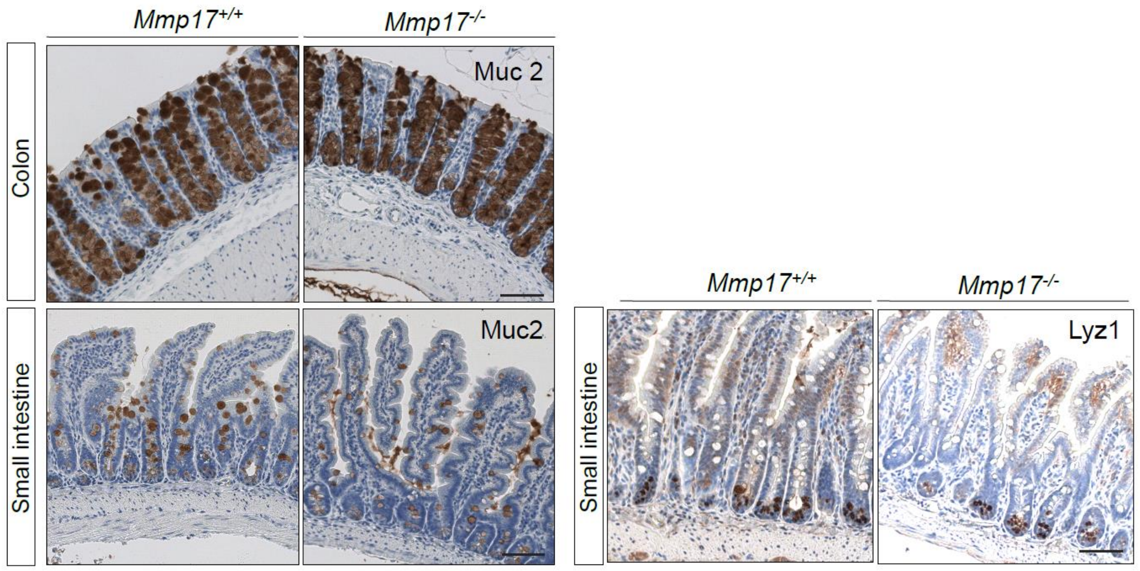

c

Enrichr
ENCODE and ChEA Consensus TFs from ChIP-X
Sorted by p-value ranking

Name	P-value	Adjusted p-value
SMAD4	0.0004016	0.04177
STAT3	0.07080	1.000
SALL4	0.1558	1.000
KAT2A	0.03088	1.000
GATA1	0.2078	1.000
EGR1	0.3556	1.000
REST	0.3307	1.000
GATA2	0.3671	1.000
NFE2L2	0.3956	1.000
BCL3	0.4425	1.000

Figure S4. No structural differences in KO smooth muscle and ENRICHR results on transcription factors differences between WT and KO crypts a. Representative H&E images of transverse colon and small intestine (SI) tissues. Scale 150 µm. n= 4 mice per genotype. Graphs represent average

values for muscularis propria thickness along the swiss roll. b. Representative images of goblet cells (Muc2) and Paneth cells (Lysozyme (Lyz1), only present in small intestine) stainings. Scale bar 100um. n= 3 animals per genotype. c.List of transcription factors altered when comparing WT and KO epithelium (Enrichr), Pajd value=0.04177, two-tailed. Data in a are means ± SD and were analyzed using Mann-Whitney test. Source data are provided as a Source Data file.

a

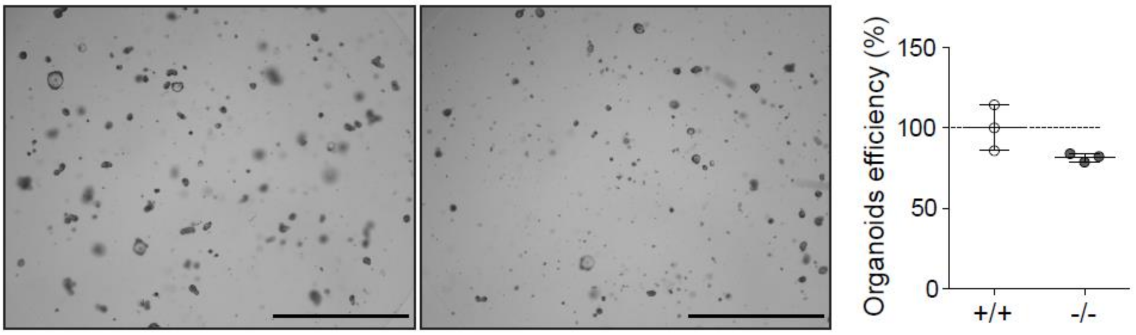

Figure S5. Organoids efficiency formation in Mmp17$^{-/-}$ small intestine cultured in ENR medium. a. Bright field representative images of small intestine organoids 72h after crypt isolation. Graph represents organoids efficiency after 72h of WT or KO crypt culture. n= 3 wells analyzed. Scale 650 µm. Data in a are means ± SD and were analyzed using Mann-Whitney test (two-sided). Source data are provided as a Source Data file.

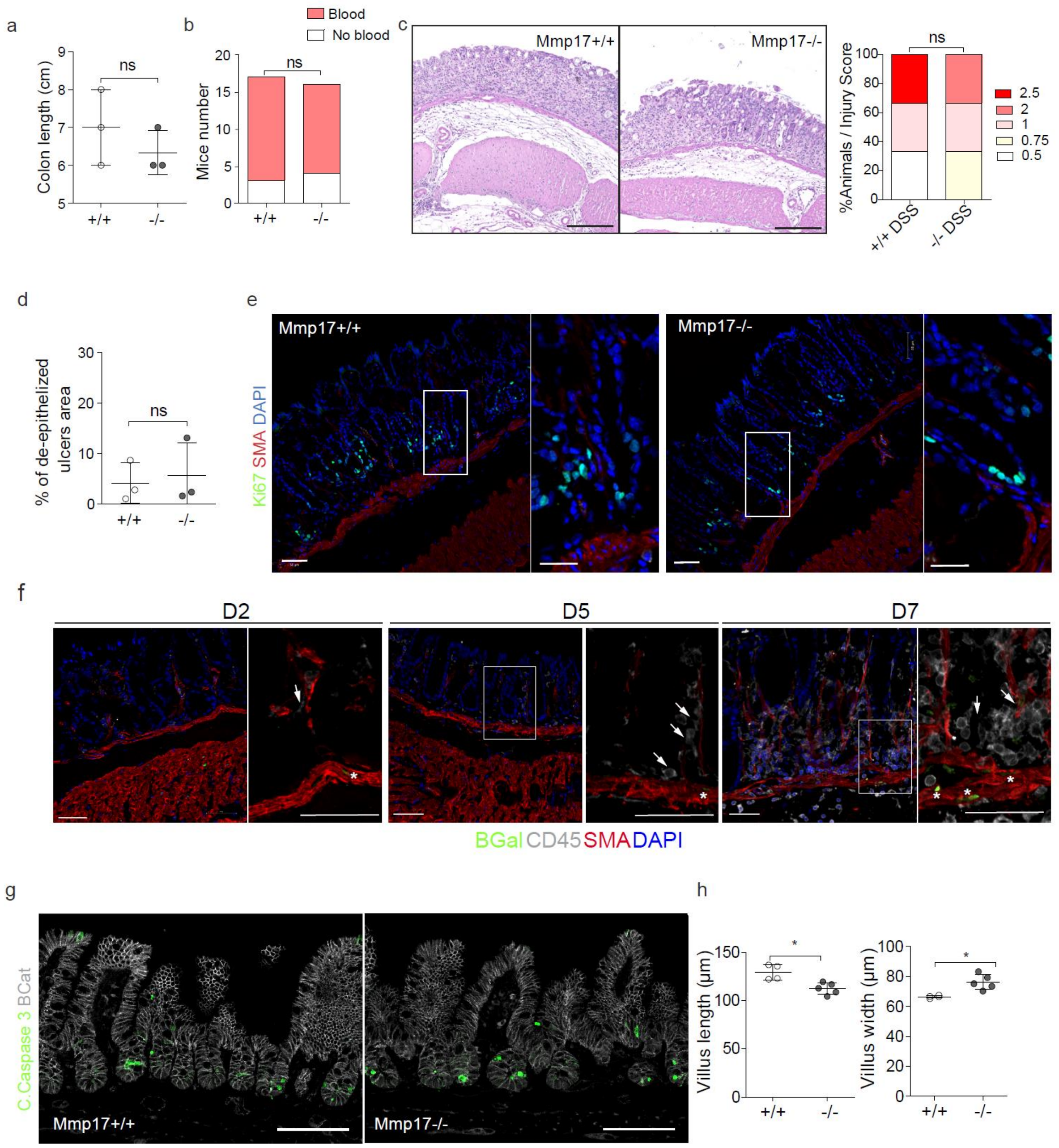

Figure S6. Intestinal damage models produce same injury level in WT and KO mice. a-d. Graphs represent colon length and presence of blood in stool at D5 of DSS treatment. n= 3 mice per genotype in a and d (1 experiment) and 17 and 16 mice in b. c. Representative H&E picture of a transverse colon cut from mice treated with DSS 3.5% for 5 days (left), and injury score evaluation of such pictures (right). Scale 200 µm. n= 3 mice per genotype, 1 experiment. d. Graph represents the presence of ulcers in the mucosa as a percentaje of the total swiss roll length at D5. n= 3 mice per genotype (1 experiment). e. Representative confocal images stained for Ki67 (green), SMA (red) and nuclei (blue), showing the equivalent reduction in proliferative cells at D5 of DSS treatment in WT and KO. Scale 50 µm; 25 µm in magnified view to the right. n= 3 mice per genotype. f. Representative confocal images showing BGal positive cells (green) during DSS timepoints treatment (Day 2, 5 and 7). BGal positive cells are SMA

(red) positive. Immune cells stained with CD45 (grey) are negative for BGal. Asterisks point to muscle BGal positive signal, arrows to immune component. Scale 50 µm. n= 2- 3 Mmp17+/- mice per time point. g. Representative confocal image showing Cleaved Caspase 3 staining (green) predominantly at the bottom of the crypts in ileum (24h after irradiation). βCat staining was performed to highlight epithelial cells. Scale bar 100 µm. n= 4 mice per genotype. h. Graph represents villi length and width in small intestinal tissue 3 days after irradiation. n= 4 WT and 5 KO mice analyzed per genotype in two independent experiments, 30 to 40 crypts/villi per mouse were quantified. Numerical data in a, d and h are means ± SD. Data were analyzed by Mann-Whitney test (a, d and h, two-sided) and one-tailed Fisher exact test in b. $p=0.0159$ in h, for both villus length and villus width. Source data are provided as a Source Data file.

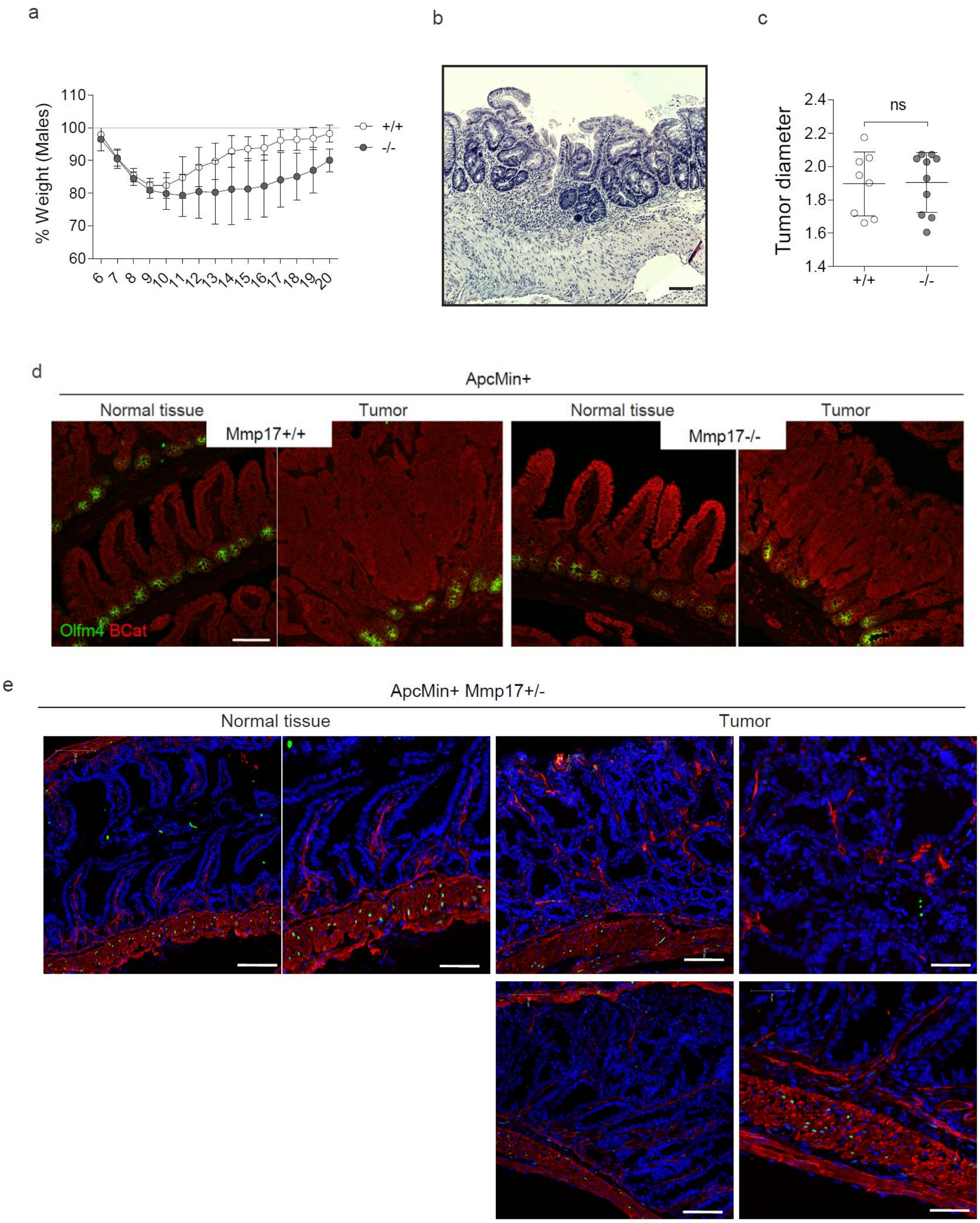

Figure S7. Mmp17 loss in ApcMin background predispose to tumors with no structural differences between WT and KO. a. Graph shows weight evolution in males after 5 days of DSS treatment. n= 3 KO and 4 WT mice per genotype. b. Representative H&E image of crypt reactive atypia in a KO mouse treated with DSS (long-term experiment). Scale bar 100 µm. n= 6-8 mice per genotype. c. Graph represents average tumor diameter (mm) in small intestines of *ApcMin+ Mmp17* WT or KO. n= 8 WT and 10 KO mice analyzed per genotype. d. Confocal maximum projection images of normal vs tumor area stained for SC marker Olfm4 (Green) and βCat (Red). Scale bar 100 µm. n= 3-5 mice

per genotype. e. Representative confocal maximum projection images showing β-Gal staining (Green) in normal tissue vs tumor areas. B-Gal signal was only found in muscle cells (SMA+, Red). Scale bar 100 µm. n= 3 mice. Data are means ± SD, numerical data in a was analyzed by two way ANOVA followed by Bonferroni post-test and t-test was applied in c. Source data are provided as a Source Data file.

a

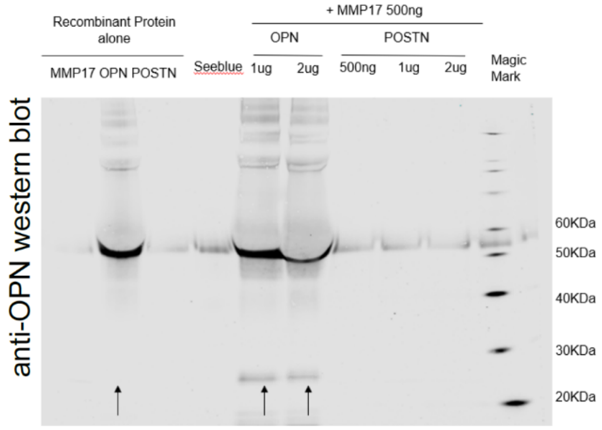

b

Peptide	Cleavage site	PSMs
RDQGPNVCALQQILGTK	R-D - 37-38	5
NVELLNALHSHMINKR	V-N - 158-159	5
AAAITSDILEALGRD	D-G - 266-267	5
FAPTNEAFEKLPR	L-F - 271-272	12
PTNEAFEK	A-P - 273-274	10
GDKVASEALMK	M-G - 291-292	7
PVTVYTTK	I-P - 664-665	11
GDGHLFEDEEIKR	G-G - 793-794	5
QGDTPVRK	L-Q - 808-809	6

c

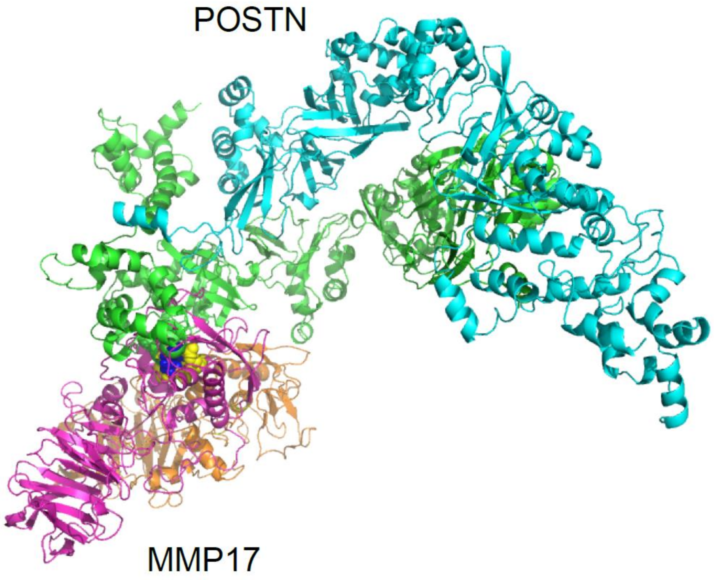

Fig. S8. MMP17 catalytic activity control. a. WB showing bands corresponding to a known MMP17 substrate named OPN (Osteopontin). Only smaller bands consistent with a cleavage product are observed when incubating the OPN together with MMP17, using the same method as for POSTN (Periostin) digestion. Full length OPN bands are observed (55KDa aprox) and cleavage products around 25KDa. An specific antibody against OPN C-terminus was used (1H3F7, Martín-Alonso et al. Cir Res. 2015). n=1 WB performed for control. b. Overview of POSTN cleavage sites with more than 5 PSMs. c. In silico model of MMP17-Periostin interaction. Model shows MMP17 dimer (magenta-orange) catalytic site in yellow in close proximity to POSTN (green-blue) cleavage site 664IP depicted in blue. Complete molecules are shown.
