# Supplementary material for: Smooth muscle-specific MMP17 (MT4-MMP) regulates the intestinal stem cell niche and regeneration after damage
Source: Nat Commun. 2021 Nov 18;12:6741. doi: 10.1038/s41467-021-26904-6 (PMC8602650; doi:10.1038/s41467-021-26904-6)
Supplement: Supplementary file 3 — Description of Additional Supplementary Files [file 41467_2021_26904_MOESM3_ESM.pdf]

Description of Additional Supplementary Files

Title: Supplementary Data 1

Description: Gene sets associated with muscle-SN treated SI organoids

Title: Supplementary Data 2

Description: List of proteins identified in muscle-SN mass spectrometry
