# Supplementary material for: Smooth muscle-specific MMP17 (MT4-MMP) regulates the intestinal stem cell niche and regeneration after damage
Source: Nat Commun. 2021 Nov 18;12:6741. doi: 10.1038/s41467-021-26904-6 (PMC8602650; doi:10.1038/s41467-021-26904-6)
Supplement: Supplementary file 6 — Reporting Summary [file 41467_2021_26904_MOESM6_ESM.pdf]

nature research

Corresponding author(s): Menno Oudhoff
Mara Martin-Alonso

Last updated by author(s): Oct 5, 2021

Reporting Summary

Nature Research wishes to improve the reproducibility of the work that we publish. This form provides structure for consistency and transparency in reporting. For further information on Nature Research policies, see our Editorial Policies and the Editorial Policy Checklist.

Statistics

For all statistical analyses, confirm that the following items are present in the figure legend, table legend, main text, or Methods section.

n/a	Confirmed	
☐	☒	The exact sample size (*n*) for each experimental group/condition, given as a discrete number and unit of measurement
☐	☒	A statement on whether measurements were taken from distinct samples or whether the same sample was measured repeatedly
☐	☒	The statistical test(s) used AND whether they are one- or two-sided<br>*Only common tests should be described solely by name; describe more complex techniques in the Methods section.*
☐	☒	A description of all covariates tested
☐	☒	A description of any assumptions or corrections, such as tests of normality and adjustment for multiple comparisons
☐	☒	A full description of the statistical parameters including central tendency (e.g. means) or other basic estimates (e.g. regression coefficient) AND variation (e.g. standard deviation) or associated estimates of uncertainty (e.g. confidence intervals)
☐	☒	For null hypothesis testing, the test statistic (e.g. *F*, *t*, *r*) with confidence intervals, effect sizes, degrees of freedom and *P* value noted<br>*Give P values as exact values whenever suitable.*
☒	☐	For Bayesian analysis, information on the choice of priors and Markov chain Monte Carlo settings
☒	☐	For hierarchical and complex designs, identification of the appropriate level for tests and full reporting of outcomes
☒	☐	Estimates of effect sizes (e.g. Cohen's *d*, Pearson's *r*), indicating how they were calculated

Our web collection on statistics for biologists contains articles on many of the points above.

Software and code

Policy information about availability of computer code

Data collection	Images were adquired with Zeiss Airyscan Software (Zen black edition) for tissue and organoids confocal images and Leica TCS SP8 STED software (Leica Application Suite) for the iECM experiments. EVOS2FL for bright field and IHC images was used. MaxQuant software (version 1.6.10.43) was used for Mass spectrometry analysis. For WB, ODYSSEY software (LI-COR Biosciences) was used.
Data analysis	In general, data have been quantified using FIJI (ImageJ), annotated in Excel, and analyzed for statistics in GraphPad Prism.<br>For RNA seq analysis, the following softwares were used: STAR was used to align reads, featureCounts was used to count reads per gene, DESeq2 was used for differential expression analysis, scikit-learn was used for principal component analysis and clusterprofiler was used for GSEA.<br>MS/MS data were searched against the Swissprot database (564277 entries, 01-2021) with Mascot.<br>For the identification of the POSTN cleavege site, proteome data (RAW files) were analyzed by Proteome Discoverer (version 2.4.1.15, Thermo Scientific) using Percolator and standard settings. For the modelled of MMP17-POSTN docking the complex was modeled using the docking protocol of the Rosetta software suite v3.12.

For manuscripts utilizing custom algorithms or software that are central to the research but not yet described in published literature, software must be made available to editors and reviewers. We strongly encourage code deposition in a community repository (e.g. GitHub). See the Nature Research guidelines for submitting code & software for further information.

Data

Policy information about availability of data

All manuscripts must include a data availability statement. This statement should provide the following information, where applicable:

- Accession codes, unique identifiers, or web links for publicly available datasets
- A list of figures that have associated raw data
- A description of any restrictions on data availability

All raw sequencing data is available through ArrayExpress. WT and KO smooth muscle and crypt RNA seq has been deposited in the ArrayExpress database under accession code E-MTAB-9180 [https://www.ebi.ac.uk/arrayexpress/experiments/E-MTAB-9180/].
The ENR vs MuscleSN treated organoids RNA seq data generated in this study has been deposited under accession code E-MTAB-9181 [https://www.ebi.ac.uk/arrayexpress/experiments/E-MTAB-9181/]. Figure 1, 2, 3 and 7 have data that comes from the RNAseq.
The mass spectrometry proteomics data have been deposited to the ProteomeXchange Consortium via the PRIDE78 partner repository with the dataset identifiers PXD020561 (MSN supernatant) http://proteomecentral.proteomexchange.org/cgi/GetDataset?ID=PXD020561 and PXD025770 (POSTN cleavage) http://proteomecentral.proteomexchange.org/cgi/GetDataset?ID=PXD025770. Figure 8 have data from the mass spectrometry experiments.
Table 2 with proteomic's data generated in this study is provided in the Supplementary Data File 2.

Field-specific reporting

Please select the one below that is the best fit for your research. If you are not sure, read the appropriate sections before making your selection.

- [x] Life sciences
- [ ] Behavioural & social sciences
- [ ] Ecological, evolutionary & environmental sciences

For a reference copy of the document with all sections, see nature.com/documents/nr-reporting-summary-flat.pdf

Life sciences study design

All studies must disclose on these points even when the disclosure is negative.

Sample size	Cohort size defined based on biological variation from pilot experiments & designed to give >80% power to detect 2-fold changes in expression patterns.<br>Sample size in organoids was determined based on biological variation in previous and related experiments.
Data exclusions	no data was excluded
Replication	We were able to replicate all results in replication experiments. Replication times are specified in each figure legend for each experiment.
Randomization	For mouse experiments littermates and/or age and sex controls were used. Mice were allocated randomly in each group.
Blinding	Histological analysis was performed blinded.

Reporting for specific materials, systems and methods

We require information from authors about some types of materials, experimental systems and methods used in many studies. Here, indicate whether each material, system or method listed is relevant to your study. If you are not sure if a list item applies to your research, read the appropriate section before selecting a response.

Materials & experimental systems

n/a	Involved in the study	
[ ]	[x]	Antibodies
[ ]	[x]	Eukaryotic cell lines
[x]	[ ]	Palaeontology and archaeology
[ ]	[x]	Animals and other organisms
[x]	[ ]	Human research participants
[x]	[ ]	Clinical data
[x]	[ ]	Dual use research of concern

Methods

n/a	Involved in the study	
[x]	[ ]	ChIP-seq
[x]	[ ]	Flow cytometry
[x]	[ ]	MRI-based neuroimaging

Antibodies

Antibodies used	Anti: Ki67 (rabbit monoclonal antibody (mAb), Invitrogen, MA5-14520), β-catenin (mouse mAb, BD Biosciences, 610154), YAP (rabbit mAb, Cell Signalling, 14074). E-Cadherin (DECMA-1 clone, Sigma, MABT26) used in iECM studies and E-Cadherin (Rabbit mAb, Cell signaling, 3195) used in intestinal swiss rolls, Muc2 (Santa Cruz, rabbit mAb, sc-515032), Lysozyme (rabbit polyclonal antibody (pAb), Dako, A0099), b-Galactosidase (βGal, Rabbit pAb, Abcam, ab4761), SMAD4 (Rabbit mAb, Cell signaling, 46535), Olfm4 (Rabbit mAb,

	Cell signaling, 39141), cleaved caspase 3 (Rabbit pAb, Cell signaling, 9661), pSMAD1/5/9 (Rabbit mAb, 13820, Cell Signaling), SMA-Cy3 directly labeled antibody (Sigma-Aldrich C6198), Desmin (Mouse mAb, Thermo Fisher Scientific ,MA5-13259) CD31, (Hamster mAb, Millipore, MAB1398z), CD45 (Rat mAb, Abcam, 25386). Periostin (Mouse mAb, SAB4200197, MERK, Sigma-Aldrich), β-tubulin (ab6160, Abcam,) and GAPDH (mouse mAb, Abcam, ab125247). The following secondary antibodies were used for immunostaining: Goat anti-Rabbit 488 (Invitrogen, A11034), Goat anti Mouse 555 (Invitrogen, A21422), Goat anti Mouse 647 (Invitrogen A21236), Goat anti Rat 647 (Invitrogen, A21247) and for WB, Goat anti Rabbit 800 (Licor, 925-32211) Goat anti mouse 680 (Licor 925-68070) and Goat anti Mouse 800 (Licor, 926-322210).
Validation	We commonly use negative controls (no primary antibody, untreated or KO sample) for all of our staining. All primary antibodies have been described before for the species and the method used, as reported in its corresponding commercial website.

Eukaryotic cell lines

Policy information about cell lines

Cell line source(s)	L-WRN (https://www.atcc.org/products/crl-3276), Rspo1 293-T cell line (kind gift from Dr. Calvin Kuo), Noggin HEK293 cell line (kind gift from Dr. Hans Clevers, L-Wnt3a (kind gift from Dr. Hans Clevers).
Authentication	We did not authenticate the cell lines
Mycoplasma contamination	Cells were confirmed negative for mycoplasma
Commonly misidentified lines (See ICLAC register)	*Name any commonly misidentified cell lines used in the study and provide a rationale for their use.*

Animals and other organisms

Policy information about studies involving animals; ARRIVE guidelines recommended for reporting animal research

Laboratory animals	Mus musculus (C57BL/5) Mmp17 LacZ/LacZ (males and females), and ApcMin (males and females) strains were used. Mice were housed in a SPF facility, with controlled temperature between 21 and 22 degrees Celsius and relative humidity between 45 and 60 %. The animals are housed in a 12 hour dark/12 hour light cycle, with 1 hour of dusk/dawn. All used mice were between 8 and 20 weeks of age.
Wild animals	no wild animals were used in the study
Field-collected samples	no field collected samples were used in the study
Ethics oversight	Norwegian Food Safety Authority, FOTS protocols (11842, 15888 and 17072). The ethical committee was constituted by Ole Aamodt and Marianne Waldum Furnes.

Note that full information on the approval of the study protocol must also be provided in the manuscript.
